# Endogenous hydrogen sulfide maintains eupnea in an in situ arterially perfused preparation of rats

**DOI:** 10.1038/s42003-020-01312-6

**Published:** 2020-10-16

**Authors:** Minako Okazaki, Saori Uozu, Yuma Sato, Masayuki Matsumoto, Tadachika Koganezawa

**Affiliations:** 1 Department of Physiology, Division of Biomedical Science, Faculty of Medicine, University of Tsukuba, Tsukuba, Ibaraki 305-8575 Japan; 2 Master’s Program in Medical Sciences, Graduate School of Comprehensive Human Sciences, University of Tsukuba, Tsukuba, Ibaraki 305-8575 Japan; 3 Transborder Medical Research Center, University of Tsukuba, Tsukuba, Ibaraki 305-8575 Japan; 4 Department of Cognitive Neuroscience, Division of Biomedical Science, Faculty of Medicine, University of Tsukuba, Tsukuba, Ibaraki 305-8575 Japan

**Keywords:** Respiration, Central pattern generators

## Abstract

Hydrogen sulfide (H_2_S) is constitutively generated in the human body and works as a gasotransmitter in synaptic transmission. In this study, we aimed to evaluate the roles of endogenous H_2_S in generating eupnea at the respiratory center. We employed an in situ arterially perfused preparation of decerebrated rats and recorded the central respiratory outputs. When the H_2_S-producing enzyme cystathionine β-synthase (CBS) was inhibited, respiration switched from the 3-phase eupneic pattern, which consists of inspiration, postinspiration, and expiration, to gasping-like respiration, which consists of inspiration only. On the other hand, when H_2_S synthesis was inhibited via cystathionine γ-lyase (CSE) or when H_2_S synthesis was activated via CBS, eupnea remained unchanged. These results suggest that H_2_S produced by CBS has crucial roles in maintaining the neuronal network to generate eupnea. The mechanism of respiratory pattern generation might be switched from a network-based system to a pacemaker cell-based system in low H_2_S conditions.

## Abstract (web summary)

Minako Okazaki et al. show that blockade of cystathionine β-synthase, which produces H_2_S gas, evoked gasping in an in situ arterially perfused preparation of decerebrated rats, whereas inhibition of cystathionine γ-lyase produced no response. These results highlight the importance of endogenous H_2_S in maintaining eupnea at the respiratory center.

## Introduction

Hydrogen sulfide (H_2_S) is generally known as a toxic gas that smells of rotten eggs. It can inhibit complex IV of mitochondria and cause dysfunction of cell respiration. If its concentration levels become too high, severe conditions such as corneal disorder, pulmonary function impairment, and respiratory paralysis may occur^1–3^. However, hydrogen sulfide is also generated in the human body mainly from l-cysteine by the pyridoxal-5′-phosphate-dependent enzymes, cystathionine γ-lyase (CSE), and cystathionine β-synthase (CBS)^2^. While CSE is located mainly in the periphery, such as in the arteries, veins, digestive tract, liver, and kidneys, CBS is located in both the periphery and the brain^4^. Recently, many studies have reported on the physiological functions of endogenously produced H_2_S, such as cytoprotection, vasodilation, and neuromodulation^1,5^. Hydrogen sulfide has been referred to as the third gaseous signaling molecule, after carbon monoxide (CO) and nitric oxide (NO). It is involved in several brain-mediated responses and regulates many body functions by changing the transmission efficiency between presynapse and postsynapse depending on the physiological conditions^6,7^.

Normally, respiration is generated by a neural network at the respiratory center in the brainstem and spinal cord^8^. The frequency and pattern of its rhythmic activity regulate oxygen and carbon dioxide levels in the body^9^. In normoxia, eupnea is usually observed and consists of three phases: inspiration, postinspiration, and expiration^10^. The neural network consisting of the pre-Bötzinger complex, the Bötzinger complex, and the pontine respiratory group is essential for eupneic rhythmogenesis, and the ablation of these regions causes alteration of the respiratory pattern^11^. This rhythmic respiratory pattern is modified depending on the physiological conditions such as the oxygen concentration. For instance, under severe hypoxia, the respiratory pattern switches from the three-phase pattern to the one-phase pattern, which is called gasping and is a powerful mechanism of autoresuscitation^12^. Given that H_2_S has roles as a neuromodulator, H_2_S might be involved in the neural network to generate respiration. Application of an H_2_S donor or inhibition of H_2_S synthesis in the respiratory center also changes the burst frequency or ventilatory outputs as shown in slice preparations or rhythmic brainstem spinal cord preparation of neonatal rats and in vivo preparation of adult rats^13–15^. This suggests that endogenous H_2_S may affect respiratory pattern generation in the brain. However, the physiological roles of endogenous H_2_S in generating the respiratory pattern by the mature central nervous system remain unknown.

In this study, we hypothesized that endogenous H_2_S is essential for maintaining eupnea generated by the complex interaction among neurons in the respiratory center. Thus, our aim was to evaluate the functional roles of endogenous H_2_S in generating the respiratory pattern at the respiratory center of juvenile rats. To attain this goal, we employed an in situ arterially perfused preparation of juvenile rats and an in vivo preparation of adult rat with modified H_2_S synthesis and observed the respiratory outputs. Here we show that when H_2_S synthesis was inhibited, eupnea was not sustained but changed to gasping. This finding indicates that the respiratory pattern-generating mechanism is maintained by H_2_S in eupnea.

## Results

### Inhibition of H_2_S synthesis via CBS by AOA and HA

**Fig. 1:**
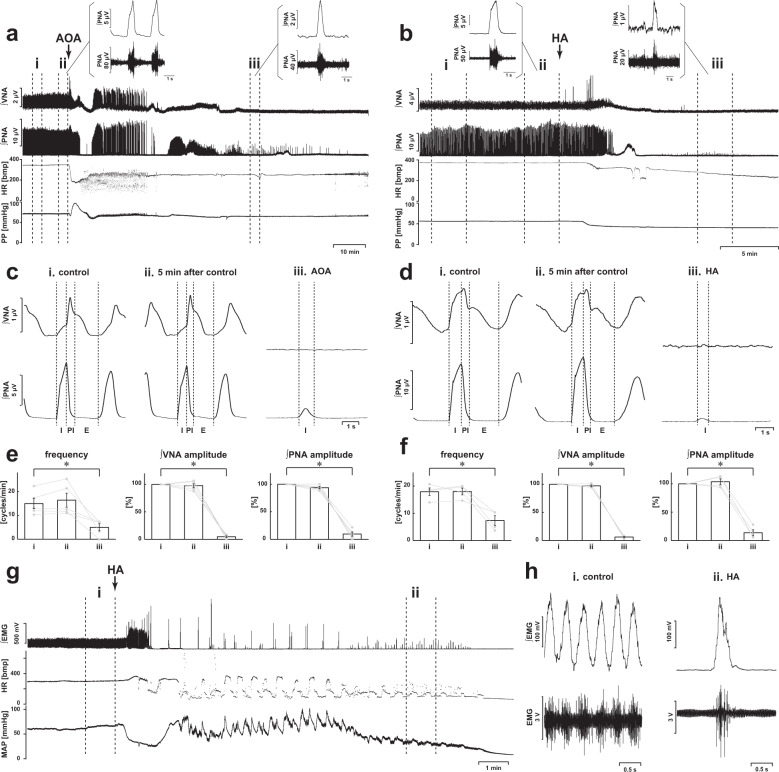
Effects of CBS inhibition on respiration. **a**, **b** Overall effects of CBS inhibition with in situ perfused preparation by AOA (**a**) and HA (**b**) on the integrated activities of the vagus nerve (∫VNA) and phrenic nerve (∫PNA), heart rate (HR), and perfusion pressure (PP). Upper figures show typical examples of PNA and ∫PNA before and after drug administration. The arrows indicate the time of drug administration. **c**, **d** Phrenic burst-triggered averages of the ∫VNA and ∫PNA after inhibition of CBS by AOA (**c**) and HA (**d**). Each waveform was obtained by averaging the ∫VNA and ∫PNA before (i and ii) and after (iii) drug administration in (**a**, **c**) and (**b**, **d**). I inspiration, PI postinspiration, E expiration. When CBS inhibitors were administered, gasping-like respiration was observed. **e**, **f** Respiratory frequencies and amplitudes of the ∫VNA and ∫PNA before (i and ii) and after (iii) administration of AOA (*n* = 5, **e**) and HA (*n* = 4, **f**). The asterisks indicate *p* < 0.05 as compared with i. Results were expressed as means ± SEMs. **g** Overall effects of CBS inhibition by HA with the in vivo experiment on the integrated activities of the diaphragmic electromyogram (∫EMG), HR, and mean arterially pressure (MAP). **h** Typical examples of ∫EMG and EMG before and after administration of HA.

To confirm the functional roles of H_2_S production via CBS in respiratory pattern generation, we observed the effects of administration of the CBS inhibitors AOA (Fig. 1a) and HA (Fig. 1b) in the in situ arterially perfused preparation. In normal conditions, the phrenic and vagus nerve activities had the rhythmic three-phase respiratory pattern, which consists of incremental activity of the phrenic and vagus nerves in the inspiratory phase, short excitation of the vagus nerve in the postinspiratory phase, and slow decrement of the vagus nerve activity in the expiratory phase (i in Fig. 1c, d). We defined this pattern as eupnea according to previous reports^12,16^.

When the CBS inhibitors were administered, the nerve activities became silent, and gasping-like respiration was observed (AOA, 4/5; HA, 4/4; iii in Fig. 1a, b). In this study, we defined gasping as short excitation of the phrenic nerve with a decremental shape and absence of expiratory vagus nerve activity (iii in Fig. 1c, d). The amplitude of both the phrenic and the vagus nerve activities and the respiratory frequency in gasping were significantly decreased when compared with those in eupnea before the administration of AOA (respiratory frequency in the control, 15.0 ± 2.2 cycles/min, with AOA, 5.0 ± 1.5 cycles/min, *p* = 0.021; amplitude of phrenic nerve activity with AOA, 9.6 ± 3.8%, *p* < 0.001 vs. control; amplitude of vagus nerve activity with AOA, 4.8 ± 1.4%, *p* < 0.001 vs. control; Fig. 1e) and HA (respiratory frequency in control, 17.9 ± 1.4 cycles/min, with HA, 7.3 ± 1.8 cycles/min, *p* = 0.001; amplitude of phrenic nerve activity with HA, 13.7 ± 5.2%, *p* < 0.001 vs. control; amplitude of vagus nerve activity with HA, 6.2 ± 1.2%, *p* < 0.001 vs. control; Fig. 1f). Administration of CBS inhibitors caused significant decrease in HR but not in PP compared with those before administration of AOA (HR in the control, 347.2 ± 4.5 bpm, with AOA, 229.5 ± 11.2 bpm, *p* < 0.001; PP in the control, 73.7 ± 8.1 mmHg, with AOA, 63.2 ± 4.8 mmHg, *p* = 0.477) or HA (HR in the control, 356.6 ± 12.8 bpm, with HA, 234.2 ± 11.9 bpm, *p* < 0.001; PP in the control, 64.1 ± 5.7 mmHg, with HA, 47.2 ± 4.8 mmHg, *p* = 0.084).

We also tested the effects of inhibition of H_2_S synthesis via CBS with HA in anesthetized rats (Fig. 1g). In normal conditions, the EMG of the diaphragm showed a rhythmic respiratory pattern with incremental activity in the inspiratory phase (i in Fig. 1h). After the administration of HA, the EMG of the diaphragm became silent in all the experiments (*n* = 5). In some experiments (2/5), gasping-like respiration, which showed decremental activity of the EMG, was observed before the respiration was completely stopped (ii in Fig. 1h).

### Effects of carotid sinus nerve denervation (CSD) on inhibition of H_2_S synthesis via CBS

**Fig. 2:**
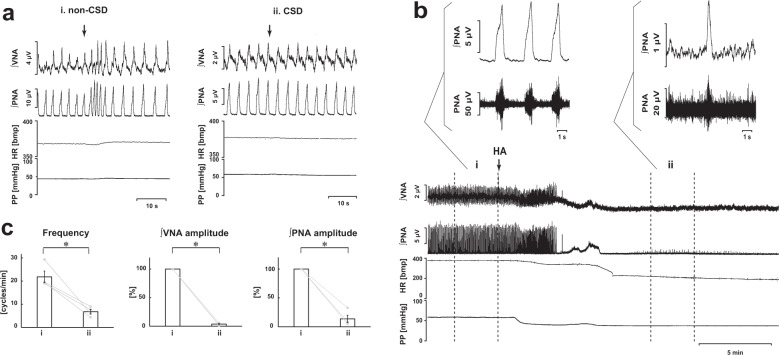
Effects of CBS inhibition on respiration in carotid sinus nerve denervated rats. **a** Effects of peripheral chemoreceptor stimulation by NaCN on the integrated activities of the vagus nerve (∫VNA) and phrenic nerve (∫PNA), heart rate (HR), and perfusion pressure (PP) in non-CSD rats (i) and CSD rats (ii). The arrows indicate the time of drug administration. **b** Overall effects of CBS inhibition with in situ perfused preparation by HA on the integrated activities of the vagus nerve (∫VNA) and phrenic nerve (∫PNA), heart rate (HR), and perfusion pressure (PP). The upper figures show typical examples of PNA and ∫PNA before and after drug administration. The arrows indicate the time of drug administration. **c** Respiratory frequencies and amplitudes of the ∫VNA and ∫PNA before (i) and after (ii) administration of HA (*n* = 4). The asterisks indicate *p* < 0.05 as compared with i. Results were expressed as means ± SEMs.

To examine whether inhibition of CBS changes the respiratory pattern generation via modification of peripheral chemoreceptor activity, we inhibited H_2_S synthesis via CBS in the in situ preparations with carotid sinus nerve denervation (CSD). In the CSD rats, chemoreflex responses in the phrenic and vagus nerves to intraaortic application of NaCN were not observed. In all the experiments with CSD rats (*n* = 4), after the administration of HA, the respiration was changed from a three-phase eupneic pattern to a gasping-like pattern, as seen in carotid sinus nerve-intact rats (Fig. 2b). The amplitude of both the phrenic and the vagus nerve activities and the respiratory frequency in gasping were significantly decreased when compared with those in eupnea before the administration of HA (respiratory frequency in the control, 21.8 ± 2.5 cycles/min, with HA, 6.8 ± 1.0 cycles/min, *p* = 0.014; amplitude of phrenic nerve activity with HA, 13.8 ± 6.4%, *p* < 0.001 vs. control; amplitude of vagus nerve activity with HA, 3.7 ± 1.2%, *p* < 0.001 vs. control; Fig. 2c).

### Blockade of persistent sodium channels by inhibition of CBS

**Fig. 3:**
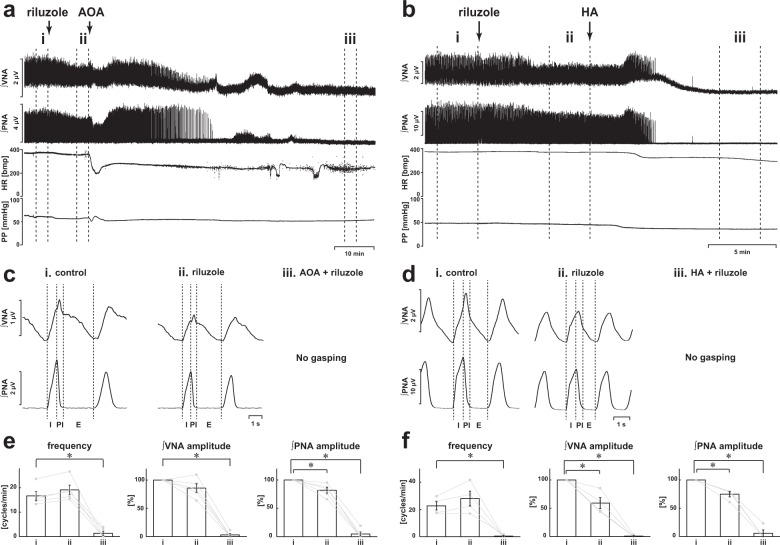
Effects of inhibition of CBS by blockade of persistent Na^+^ channels. **a**, **b** Overall effects of blockade of persistent Na^+^ channels by riluzole and inhibition of CBS by AOA (**a**) and HA (**b**) on the integrated activities of the vagus nerve (∫VNA) and phrenic nerve (∫PNA), heart rate (HR), and perfusion pressure (PP). The arrows indicate the time of drug administration. **c**, **d** Phrenic burst-triggered averages of the ∫VNA and ∫PNA after inhibition of CBS by AOA (**c**) and HA (**d**) in the presence of riluzole. Each waveform was obtained by averaging the ∫VNA and ∫PNA before administration of riluzole (i) and CBS inhibitors (ii), and after administration of the drugs (iii) in (**a**, **c**) and (**b**, **d**). In the presence of riluzole, gasping-like respiration was not observed after administration of CBS inhibitors. **e**, **f** Respiratory frequencies and amplitudes of the ∫VNA and ∫PNA before administration of riluzole (i) and CBS inhibitors (ii) and after administration of both riluzole and CBS inhibitors (iii; AOA, *n* = 5, **e**; HA, *n* = 4, **f**). The asterisks indicate *p* < 0.05 as compared with i. Results were expressed as means ± SEMs.

**Fig. 4:**
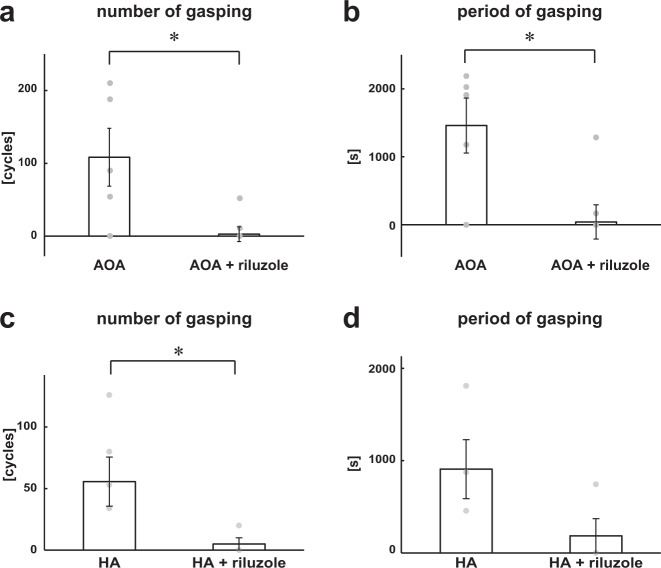
Gasping-like respiration after inhibition of CBS and blockade of persistent Na^+^ channels. **a**, **b** Number (**a**) and period (**b**) of gasping-like respirations after administration of AOA only (*n* = 5) or of both AOA and riluzole (*n* = 5). The asterisks indicate *p* < 0.05. Results were expressed as means ± SEMs. Both the number and the period of AOA-induced gasping were significantly suppressed in the presence of riluzole. **c**, **d** Number (**c**) and period (**d**) of gasping-like respirations after administration of HA only (*n* = 4) or of both HA and riluzole (*n* = 4). The asterisks indicate *p* < 0.05. Results were expressed as means ± SEMs. The number of HA-induced gasps was significantly suppressed in the presence of riluzole.

Next, we tested the effect of riluzole, which blocks the persistent sodium channels in medullary respiratory neurons^12^, on AOA- (Fig. 3a) and HA- (Fig. 3b) induced respiratory changes. Even in the presence of riluzole, the three-phase-based eupneic pattern was maintained (ii in Fig. 3c, d), although the amplitude of the phrenic and vagus nerve activities was reduced in the presence of riluzole without AOA (respiratory frequency in the control, 16.7 ± 1.8 cycles/min, with riluzole, 19.1 ± 2.0 cycles/min, *p* = 0.481; amplitude of phrenic nerve activity with riluzole, 81.9 ± 5.0%, *p* = 0.007 vs. control; amplitude of vagus nerve activity with riluzole, 85.8 ± 7.6%, *p* = 0.088 vs. control; Fig. 3e) and HA (respiratory frequency in control, 22.7 ± 3.1 cycles/min, with riluzole, 28.0 ± 5.4 cycles/min, *p* = 0.498; amplitude of phrenic nerve activity with riluzole, 74.9 ± 4.6%, *p* = 0.005 vs. control; amplitude of vagus nerve activity with riluzole, 59.3 ± 9.7%, *p* = 0.001 vs. control; Fig. 3f). Neither HR nor PP was affected by riluzole in the absence of AOA (HR in the control, 352.5 ± 6.9 bpm, with riluzole, 338.3 ± 5.6 bpm, *p* = 0.266; PP in the control, 69.7 ± 4.6 mmHg, with riluzole, 62.4 ± 2.8 mmHg, *p* = 0.249) or HA (HR in the control, 368.3 ± 4.5 bpm, with riluzole, 356.2 ± 7.4 bpm, *p* = 0.818; PP in the control, 45.7 ± 1.3 mmHg, with riluzole, 41.3 ± 1.6 mmHg, *p* = 0.148). When AOA or HA was administered in the presence of riluzole, the gasping-like respiration was attenuated (AOA, 2/5; HA, 1/4) or not observed (AOA, 3/5; HA, 3/4; iii in Fig. 3c, d). The amplitude of both the phrenic and the vagus nerve activities and the respiratory frequency in gasping-like respiration were significantly suppressed when compared with those in eupnea in the absence of both riluzole and AOA (respiratory frequency in control, 16.7 ± 1.8 cycles/min, with AOA and riluzole, 1.4 ± 0.9 cycles/min, *p* < 0.001; amplitude of phrenic nerve activity with AOA and riluzole, 4.8 ± 3.7%, *p* < 0.001 vs. control; amplitude of vagus nerve activity with AOA and riluzole, 3.0 ± 2.3%, *p* < 0.001 vs. control; Fig. 3e) or HA (respiratory frequency in control, 22.7 ± 3.1 cycles/min, with HA and riluzole, 0.4 ± 0.4 cycles/min, *p* = 0.007; amplitude of phrenic nerve activity with HA and riluzole, 5.9 ± 5.9%, *p* < 0.001 vs. control; amplitude of vagus nerve activity with HA and riluzole 0.8 ± 0.8%, *p* < 0.001 vs. control; Fig. 3f). HR and PP were also significantly decreased when compared with HR in the absence of both riluzole and AOA (HR in the control, 352.5 ± 6.9 bpm, with riluzole, 338.3 ± 5.6 bpm, *p* = 0.266; PP in the control, 69.7 ± 4.6 mmHg, with riluzole, 62.4 ± 2.8 mmHg, *p* = 0.249) or HA (HR in the control, 368.3 ± 4.5 bpm, with riluzole and HA, 249.3 ± 26.4 bpm, *p* = 0.001; PP in the control, 45.7 ± 1.3 mmHg, with riluzole and HA, 31.4 ± 1.8 mmHg, *p* < 0.001). The number of gasps was significantly lower than that when AOA or HA alone was administered (AOA alone, 108.4 ± 39.8 cycles, AOA and riluzole, 2.8 ± 10.1 cycles, *p* = 0.048; HA alone, 55.7 ± 20.0 cycles, HA and riluzole, 5.0 ± 5.0 cycles, *p* = 0.016) (Fig. 4).

### Inhibition of H_2_S synthesis via CSE

**Fig. 5:**
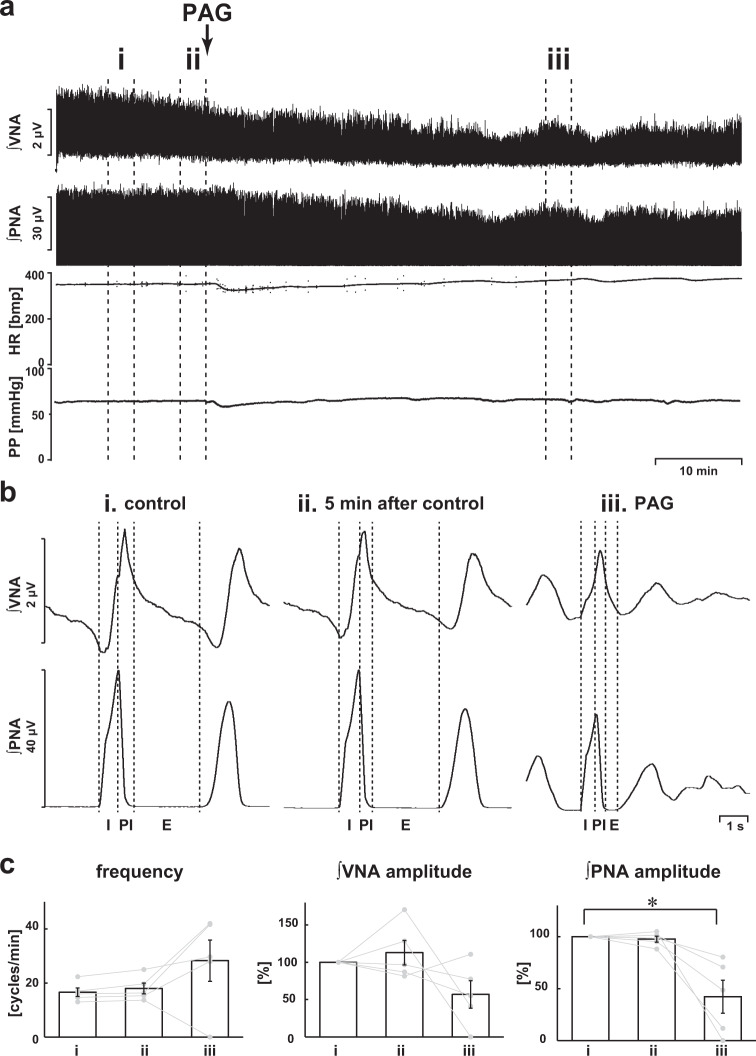
Effects of inhibition of CSE by PAG on respiration. **a** Overall effects of the inhibition of CSE by PAG on the integrated activities of the vagus nerve (∫VNA) and phrenic nerve (∫PNA), heart rate (HR), and perfusion pressure (PP). The arrow indicates the time of PAG administration. **b** Phrenic burst-triggered averages of the ∫VNA and ∫PNA after inhibition of CSE by PAG. Each waveform was obtained by averaging the ∫VNA or ∫PNA before (i and ii) and after (iii) administration of PAG in (**a**). I inspiration, PI postinspiration, E expiration. Even after PAG administration, the eupneic pattern was maintained. **c** Respiratory frequencies and amplitudes of the ∫VNA and ∫PNA before (i and ii) and after (iii) PAG administration (*n* = 5). The asterisks indicate *p* < 0.05 as compared with i. Results were expressed as means ± SEMs.

We also evaluated the involvement of CSE, another H_2_S synthesis enzyme, in generating eupnea. When PAG, which is a CSE inhibitor, was administered, the respiratory pattern was not changed and the three-phase rhythmic pattern was maintained, although the amplitude of phrenic nerve activity was significantly decreased when compared with those in the absence of PAG (respiratory frequency in control, 16.6 ± 1.6 cycles/min, with PAG, 28.3 ± 7.6 cycles/min, *p* = 0.174; amplitude of phrenic nerve activity with PAG, 42.3 ± 15.8%, *p* = 0.002 vs. control; amplitude of vagus nerve activity with PAG, 56.8 ± 18.5%, *p* = 0.096 vs. control; Fig. 5). HR and PP remained constant when the absence of PAG was compared with the presence of it (HR in the control, 344.8 ± 9.4 bpm, with PAG, 327.6 ± 25.4 bpm, *p* = 0.695; PP in the control, 53.2 ± 5.1 mmHg, with PAG, 52.6 ± 6.1 mmHg, *p* = 0.995).

### Activation of H_2_S synthesis via CBS

**Fig. 6:**
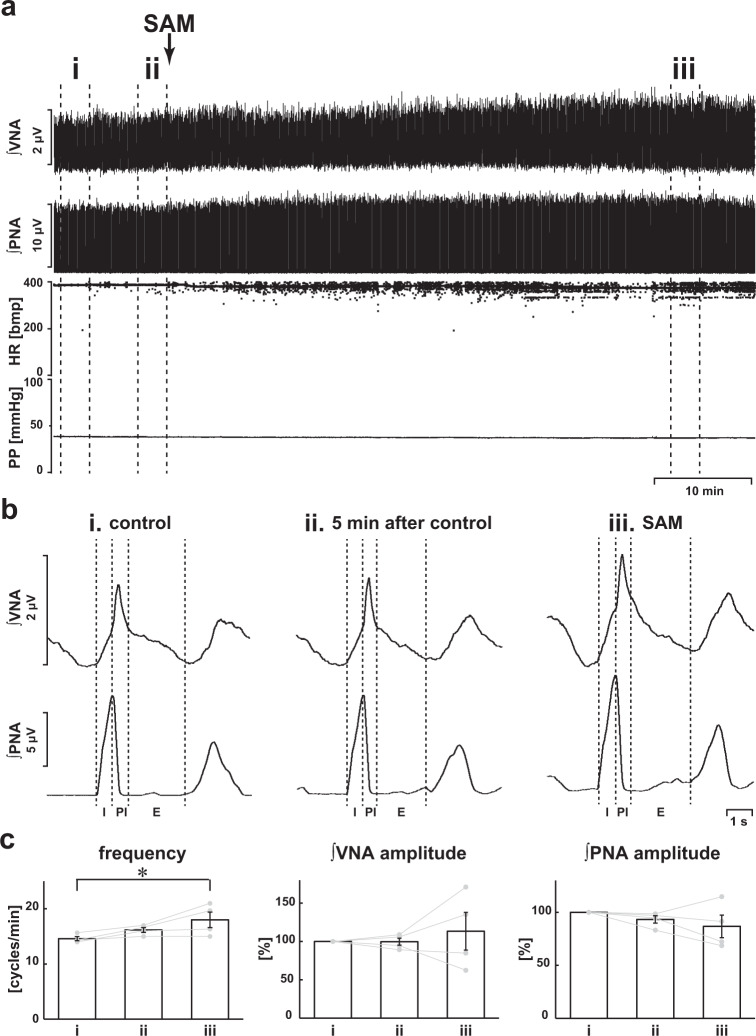
Effects of activation of CBS by SAM on respiration. **a** Overall effects of activation of CBS by SAM on the integrated activities of the vagus nerve (∫VNA) and phrenic nerve (∫PNA), heart rate (HR), and perfusion pressure (PP). The arrow indicates the time of SAM administration. **b** Phrenic burst-triggered averages of the ∫VNA and ∫PNA after activation of CBS by SAM. Each waveform was obtained by averaging the ∫VNA or ∫PNA before (i and ii) and after (iii) SAM administration in (**a**). I inspiration, PI postinspiration, E expiration. Even after SAM administration, the eupneic pattern was maintained. **c** Respiratory frequencies and amplitudes of the ∫VNA and ∫PNA before (i and ii) and after (iii) SAM administration (*n* = 4). The asterisks indicate *p* < 0.05 as compared with i. Results were expressed as means ± SEMs.

We also examined the effect of activation of H_2_S synthesis from administration of S-adenosyl-l-methionine (SAM), which is a CBS activator. Even in the presence of SAM, although the respiratory frequency was significantly increased, the eupneic respiratory pattern was maintained and the amplitude of both the phrenic and vagus nerve activities and the respiratory frequency were not statistically changed (respiratory frequency in control, 14.6 ± 0.4 cycles/min, with SAM, 18.0 ± 1.4 cycles/min, *p* = 0.040; amplitude of phrenic nerve activity with SAM, 86.7 ± 10.6%, *p* = 0.292 vs. control; amplitude of vagus nerve activity with SAM, 113.4 ± 24.5%, *p* = 0.742 vs. control; Fig. 6). HR and PP did not change with administration of SAM (HR in the control, 365.7 ± 7.3 bpm, with SAM, 360.7 ± 4.9 bpm, *p* = 0.807; PP in the control, 54.2 ± 10.4 mmHg, with SAM, 53.5 ± 9.1 mmHg, *p* = 0.998).

## Discussion

In an in situ arterially perfused preparation of rats, inhibition of H_2_S production via CBS changed eupnea into gasping, which was suppressed by the presence of a persistent sodium channel blocker. On the other hand, inhibition of CSE and activation of CBS did not change the eupneic respiratory pattern. These findings suggest that CBS-synthesized H_2_S has a functional role in maintaining the network-based respiratory pattern generation in eupnea by sustaining synaptic transmission and that reduction of endogenous H_2_S in the brain switches the pattern generation into a pacemaker-based system by attenuating synaptic transmission.

Eupnea, which is generated by a complex neural network in the respiratory center, is composed of three phases: inspiration, postinspiration, and expiration^17^. This three-phase pattern is modified depending on the functional connectivity among neurons. For instance, it has been reported that central severe hypoxia, which attenuates synaptic transmission, or transection of the pons causes switching of the respiratory pattern from the three-phase pattern to the one-phase pattern^12,18^. However, the involvement of H_2_S, which is known as a synaptic modulator, in generating rhythmic respiration remains unknown. In this study, inhibition of H_2_S synthesis via CBS caused switching of the respiratory pattern from the three-phase pattern, eupnea, to the one-phase pattern with decremental nerve activity, gasping. We also used urethane-anesthetized rats and confirmed that the respiratory pattern was changed into gasping by the CBS inhibitor, similarly to those in the perfused preparation. This result supports the notion that CBS inhibition surely causes the change in the respiratory pattern. In the urethane-anesthetized rats, gasping was observed only in two out of five rats before the respiration was completely stopped. It has been also reported that, severe hypoxemia little induces gasping in urethane-anesthetized rats but it causes gasping in ketamine/xylazine and pentobarbital anesthetized rats^19^. Therefore, the lack of gasping in three out of five rats might be caused by a specific effect of urethane but not by a functional difference between the anesthetized and the perfused preparations. Moreover, administration of riluzole, a persistent sodium current blocker, suppressed CBS inhibitor-induced gasping but not eupnea. The persistent sodium current is an important component of pacemaker properties in respiratory-pacemaker neurons^20–24^. Therefore, this result suggests that the respiratory pattern-generating mechanism is switched from a network-based system to a pacemaker cell-based system by the inhibition of H_2_S production via CBS. It has been reported that H_2_S has a role as a neuromodulator and can modulate the transmission efficiency^1^. Under eupnea, at the respiratory center, H_2_S may facilitate the transmission efficiency and potentiate the neural network to generate respiration. However, when the H_2_S level is decreased, attenuation of the transmission efficiency and the connectivity of the network may induce switching to pacemaker-based generation of respiration. It was previously reported that central severe hypoxia, which attenuates synaptic transmission, causes the switching of the respiratory pattern from eupnea to gasping^11,12,25^. By the application of riluzole, eupnea was little affected and gasping was suppressed. This result indicates that respiratory pattern generation was switched to the pacemaker-based system. Our present study of CBS inhibition showed similar results as those for severe hypoxia. In other words, the switching of respiratory pattern generation may underlie the change of the respiratory pattern after CBS inhibition in the same way as with central severe hypoxia. Thus, H_2_S produced by CBS surely has a crucial role in maintaining eupneic-pattern respiration.

To confirm involvement of endogenous H_2_S in peripheral chemoreceptors in maintenance of eupnea, we employed CSD rats and observed effects on the changes in the respiratory pattern with an CBS inhibitor. The results showed that even under CSD conditions, administration of an CBS inhibitor caused the same changes in the respiratory pattern as those in CSD-intact rats. This result indicates that centrally generated H_2_S is important for maintenance of eupnea.

We also inhibited another synthetic enzyme of endogenous H_2_S, CSE. Even in the presence of the CSE inhibitor PAG, eupneic three-phase respiration was maintained, although the amplitude of the respiratory nerve activities was attenuated. The concentration of PAG was determined on the basis of previous reports, and it was high enough to inhibit CSE activity^2,26^. This suggests that H_2_S produced by CSE does not affect the continuation of the eupneic pattern. Most of the relevant studies have reported that CBS is expressed in the brain, whereas CSE is expressed only in the peripheral organs such as the arteries, veins, digestive tract, liver, and kidneys^1–3^. The present study’s findings indicate that endogenous H_2_S in the central nervous system but not in the peripheral organs has a crucial role in maintaining the eupneic respiratory pattern. The nerve activities were decreased by inhibition of CSE-dependent H_2_S production. It has been reported that CSE is located in the vascular endothelium and that synthesized H_2_S works as a vasodilator^3,27,28^. Therefore, inhibition of CSE may cause suppression of endogenous H_2_S production in the blood vessels and vasoconstriction in the brain. Thus, the slight reduction of perfusion in the brain affected the maintenance of neuronal firing in the brain. In other words, the effect of CSE inhibition was a result of the reduced amount of perfusion in the respiratory center rather than of direct effects to the neural connection due to the shortage of H_2_S produced by CSE. In fact, when H_2_S production was inhibited via CSE, the amplitude of respiratory nerve activities was attenuated, but the eupneic three-phase respiratory pattern was maintained.

In this study, we used AOA and HA as CBS inhibitors because these drugs are typically used. However, the selectivity of these drugs for CBS is not ideal^29^. AOA and HA inhibit PLP-dependent enzymes and heme-containing enzymes, respectively, and they might affect not only CBS but also other enzymes. For instance, higher concentration of these drugs causes neuronal damage, although the side effect is usually observed much longer time (>3 h) after application than our experimental protocol^30^. Moreover, it has been reported that HA and AOA also inhibit CSE^29^. However, we can ignore the nonspecific effects of those drugs on CSE because selective inhibition via PAG revealed that CSE-produced H_2_S does not have a functional role in the maintenance of eupnea. Moreover, to carefully ensure the effects of those drugs, we used two kinds of CBS inhibitors and observed constant and reproducible results.

Another enzyme, 3-mercaptopyruvate sulfurtransferase (3MST), has been reported as a novel H_2_S-producing enzyme^31^. In this study, inhibition of only CBS strongly changed the respiratory pattern, indicating that CBS-produced endogenous H_2_S in the respiratory center may have essential roles in maintaining the neural network in eupnea. However, 3MST is also localized in neurons and astrocytes^32,33^. Therefore, the possibility exists that 3MST-produced H_2_S also has functional roles in respiratory pattern generation in the respiratory center.

To evaluate the effect of activation of H_2_S production via CBS, we administered a CBS activator, SAM. In aerobic conditions, the basal concentration of H_2_S is maintained at a low level^34–36^. Moreover, the concentration of SAM that was used in this study is enough to activate CBS in the brain^37^. Therefore, activation of CBS seems to increase the endogenous H_2_S level. However, in the presence of the CBS activator, there was no change in the central respiratory outputs. This suggests that the basal level of H_2_S is already enough to maintain the neural network in the respiratory center and that endogenously generated excess H_2_S does not have any additional effects in generating the respiratory pattern.

In this study, we used an in situ arterially perfused preparation of juvenile rats^38^. Some studies have reported the involvement of exogeneous and endogenous H_2_S in respiratory regulation by the central nervous system in neonatal slice preparations, whose neural network is immature and only a part of the respiratory center in the brainstem. Actually, the central respiratory outputs of neonates in vivo and in vitro differ from those of adults^39^. Therefore, to observe the respiratory pattern switching especially from eupnea with incremental activities to gasping with decremental activities, it was necessary to perform the experiment on animals in which the whole mature respiratory center is included in the brainstem. The arterially perfused preparation was based on juvenile rats whose neural network for respiration is matured and preserved and we can observe the similar respiratory pattern switching to the experiment in vivo by severe hypoxia^40^. Also, the oxygen level in the perfusate could be maintained artificially even if the central respiratory outputs changed in the perfused preparation, while those in the in vivo preparation were modified depending on the respiration. Therefore, the arterially perfused preparation had a great advantage in terms of revealing the physiological function of endogenous H_2_S.

The respiratory center is widely spread in the spinal cord and pons, and the responsible region for each phase has been reported^11^. However, which part of the respiratory center is affected by H_2_S remains unknown. In the present study, drugs that modulate H_2_S production affected the preparation systemically because we administered them into the perfusate. Further study observing the site-specific response to H_2_S inhibition by use of local injection of inhibitors would reveal the functional sites of H_2_S.

In conclusion, H_2_S generated by CBS in the brain may have crucial roles in maintaining the eupneic pattern, which is the rhythmic three-phase respiratory pattern. Therefore, reduction of endogenous H_2_S level in the brain switches the respiratory pattern from eupnea to gasping, which is generated by a pacemaker cell-based system.

## Methods

All procedures were approved by the ethics review committee of the University of Tsukuba.

### In situ arterially perfused preparation

The experiments were performed on 35 Wistar rats (male, 3–4 weeks; Japan SLC, Inc.) according to previously described methods^38^. Briefly, the rats were intraperitoneally injected with heparin (100 U) to prevent thrombosis and deeply anesthetized with isoflurane until respiration was stopped. The rats were bisected subdiaphragmatically and transferred into iced artificial cerebrospinal fluid (ACSF). The composition of the ACSF was as follows: NaCl (125 mM), NaHCO_3_ (25 mM), KCl (5 mM), CaCl_2_ (2.5 mM), MgSO_4_ (1.25 mM), KH_2_PO4 (1.25 mM), and d-Glucose (10 mM). The cerebrocortex, cerebellum, digestive tract, and lungs were removed, and the descending aorta was isolated. The left phrenic nerve and left vagus nerve were isolated from the connective tissues of the thorax and neck, respectively. In the carotid sinus nerve-denervated group (*n* = 4), the carotid sinus nerves were bilaterally cut at the neck according to a surgical approach, as described previously^41–43^. After transferral to a recording chamber, the descending aorta was cannulated with a double-lumen catheter and the rats were perfused retrogradely with 200 mL of perfusate, which contained 1.25% polyethylene glycol in ACSF to maintain the oncotic pressure, using a roller pump at constant speed (16.0–28.4 mL/min) and temperature (31 °C). The perfusion pressure (PP) was recorded from the second lumen of the double-lumen catheter. The perfusate was constantly gassed with 95% oxygen/5% carbon dioxide. After perfusion was started, a vasoconstrictor peptide (arginine vasopressin, 0.4 nM) and a neuromuscular blocker (vecuronium bromide, 4 mg/L) were added to the perfusate to increase the peripheral vascular resistance and to abolish EMG activity, respectively.

### Electrocardiography and peripheral nerve recording

The left phrenic and left central vagus nerve activities were recorded via glass suction electrodes. These activities were amplified (×10,000) and filtered (100–5000 Hz). To obtain integrated waveforms of the nerve activities, they were rectified and smoothed by applying the moving average (0.1-s time window). The PP was monitored via a double-lumen cannula inserted into the descending aorta. The electrocardiogram (ECG) was recorded and the heart rate (HR) was calculated on the basis of the interval between two R waves.

### In vivo preparation of anesthetized rats

The experiments were performed on 4 Wistar rats (male, 310–330 g; Japan SLC, Inc.). The rats were anesthetized with urethane (1 g/kg, 250 mg/mL, *i.p*.). Depth of anesthesia was assured by loss of eyelid reflex and responses to pinching the toe, and these effects remained throughout the experiment. After the administration of urethane, preparatory surgery was performed to insert a catheter, which was filled with heparinized saline (200 IU/mL), into the femoral artery to monitor the arterial pressure directly, and the mean arterial pressure was calculated. The diaphragmic electromyogram (EMG) was recorded via wire electrodes. This activity was amplified (×10,000) and filtered (150–1000 Hz). To obtain integrated waveforms of the EMG, they were rectified and smoothed by application of the moving average (0.05-s time window). The ECG was recorded, and the HR was calculated on the basis of the ECG recording.

### Drug administration

In the in situ arterially perfused preparation, after the nerve activities became stable, they were recorded for about 11 min. Then, H_2_S synthesis was pharmacologically modulated by use of a CSE inhibitor (dl-propargylglycine [PAG], 10 mM, *n* = 5), CBS inhibitors (aminooxyacetic acid hemihydrochloride, [AOA], 10 mM, *n* = 10; hydroxylamine hydrochloride [HA], 2 mM, *n* = 8), and a CBS activator (S-adenosyl-l-methionine chloride dihydrochloride [SAM], 60 μM, *n* = 4). The concentration of those drugs was selected on the basis of previous reports^2,14,15,26,37^. The nerve activities were recorded until they became stable or silent. Furthermore, the groups treated with AOA or HA were divided into two groups: those treated with and those treated without riluzole, which blocks the persistent sodium current in medullary respiratory neurons (AOA alone, *n* = 5; AOA with riluzole, *n* = 5; HA alone, *n* = 4; HA with riluzole, *n* = 4)^12^. For the riluzole-treated groups, riluzole (5 μM) was administered into the perfusate after 3-min recording of the stable nerve activities as the control condition. Then, the preparation was left for about 5 min. After 3-min recording in the riluzole-treated condition without CBS inhibitors, AOA or HA was administered into the perfusate. The nerve activities were recorded until they became stable or silent. The administered drugs were dissolved in distilled water. The total volume of the applied solution was less than 1.2 mL in each rat, and the same volume of the vehicle, distilled water, did not affect the respiratory outputs. To stimulate peripheral chemoreceptors in the in situ preparation, NaCN (0.03%, 50 mL) was applied by an intra-aortic injection via the side-port of the aortic perfusion catheter.

In the anesthetized rats, after the respiration was stable, the diaphragmic EMG was recorded for at least 3 min. Then, HA (100–150 mg/kg, *i.v*.) was applied from the femoral vein.

### Data analysis

Outputs from the nerves, ECG, and PP were recorded directly onto a computer hard drive through an AD converter (1401-plus; Cambridge Electronic Design, Cambridge, UK) and analyzed using a data capture and analysis software (Spike2; Cambridge Electronic Design). To analyze the respiratory pattern of the phrenic and vagus nerve activities, phrenic burst-triggered averaging was applied in the integrated nerve recordings for 3 min in each condition. The amplitude of the nerve activity was measured from the averaged waveform. The respiratory frequency was also calculated according to the frequency of the inspiratory phrenic nerve activity for 3 min in each condition. When gasping-like respiration was observed for less than 3 min, the amplitude and frequency were calculated from the entire duration of gasping-like respiration. For standardization among the rats, the nerve activities in the control condition of each animal were determined as 100% and the amplitude of the responses was expressed as the percentage change.

### Statistics and reproducibility

Numerical data were expressed as means ± SEMs. To compare two different groups statistically, we used the *t* test. To compare the control condition with the other conditions, we used ANOVA with repeated measures followed by the Dunnett test. The level of significance was taken as *p* < 0.05. We repeated at least four times experiments and the exact sample size for each experiment appear in figure legend.

### Reporting summary

Further information on research design is available in the Nature Research Reporting Summary linked to this article.

## Supplementary information

Description of Additional Supplementary Files

Supplementary Data 1

Reporting Summary

## Data Availability

The data that support the findings of this study are available from the corresponding author upon reasonable request. All source data underlying the graphs and the charts presented in the main figures are available in Supplementary Data 1.

## Code availability

All codes for processing the data presented in this work are available on reasonable request.
