# Supplementary material for: Endogenous hydrogen sulfide maintains eupnea in an in situ arterially perfused preparation of rats
Source: Commun Biol. 2020 Oct 16;3:583. doi: 10.1038/s42003-020-01312-6 (PMC7568547; doi:10.1038/s42003-020-01312-6)
Supplement: Supplementary file 1 — Description of Additional Supplementary Files [file 42003_2020_1312_MOESM1_ESM.pdf]

Description of Additional Supplementary Files

File Name: Supplementary Data 1
Description: All source data underlying the graphs and charts presented in the main figures.
