# Supplementary material for: Endogenous hydrogen sulfide maintains eupnea in an in situ arterially perfused preparation of rats
Source: Commun Biol. 2020 Oct 16;3:583. doi: 10.1038/s42003-020-01312-6 (PMC7568547; doi:10.1038/s42003-020-01312-6)
Supplement: Supplementary file 3 — Reporting Summary [file 42003_2020_1312_MOESM3_ESM.pdf]

nature research

Corresponding author(s): Tadachika Koganezawa
Last updated by author(s): Sep 15, 2020

Reporting Summary

Nature Research wishes to improve the reproducibility of the work that we publish. This form provides structure for consistency and transparency in reporting. For further information on Nature Research policies, see our Editorial Policies and the Editorial Policy Checklist.

Statistics

For all statistical analyses, confirm that the following items are present in the figure legend, table legend, main text, or Methods section.

n/a	Confirmed	
☐	☒	The exact sample size (n) for each experimental group/condition, given as a discrete number and unit of measurement
☐	☒	A statement on whether measurements were taken from distinct samples or whether the same sample was measured repeatedly
☐	☒	The statistical test(s) used AND whether they are one- or two-sided<br>*Only common tests should be described solely by name; describe more complex techniques in the Methods section.*
☒	☐	A description of all covariates tested
☐	☒	A description of any assumptions or corrections, such as tests of normality and adjustment for multiple comparisons
☐	☒	A full description of the statistical parameters including central tendency (e.g. means) or other basic estimates (e.g. regression coefficient) AND variation (e.g. standard deviation) or associated estimates of uncertainty (e.g. confidence intervals)
☐	☒	For null hypothesis testing, the test statistic (e.g. F, t, r) with confidence intervals, effect sizes, degrees of freedom and P value noted<br>*Give P values as exact values whenever suitable.*
☒	☐	For Bayesian analysis, information on the choice of priors and Markov chain Monte Carlo settings
☒	☐	For hierarchical and complex designs, identification of the appropriate level for tests and full reporting of outcomes
☒	☐	Estimates of effect sizes (e.g. Cohen's d, Pearson's r), indicating how they were calculated

Our web collection on statistics for biologists contains articles on many of the points above.

Software and code

Policy information about availability of computer code

Data collection	Data was recorded using a data capture and analysis software (Spike2 version 9; Cambridge Electronic Design).
Data analysis	Raw data was analyzed using a data capture and analysis software (Spike2 version 9; Cambridge Electronic Design). Numerical data was statistically analyzed using Excel 2019 (Microsoft) or SPSS statistics 25 (IBM).

For manuscripts utilizing custom algorithms or software that are central to the research but not yet described in published literature, software must be made available to editors and reviewers. We strongly encourage code deposition in a community repository (e.g. GitHub). See the Nature Research guidelines for submitting code & software for further information.

Data

Policy information about availability of data

All manuscripts must include a data availability statement. This statement should provide the following information, where applicable:

- Accession codes, unique identifiers, or web links for publicly available datasets
- A list of figures that have associated raw data
- A description of any restrictions on data availability

The data that support the findings of this study are available from the corresponding author upon reasonable request. All source data underlying the graphs and the charts presented in the main figures are available in Supplementary Data 1.

Field-specific reporting

Please select the one below that is the best fit for your research. If you are not sure, read the appropriate sections before making your selection.

- [x] Life sciences
- [ ] Behavioural & social sciences
- [ ] Ecological, evolutionary & environmental sciences

For a reference copy of the document with all sections, see nature.com/documents/nr-reporting-summary-flat.pdf

Life sciences study design

All studies must disclose on these points even when the disclosure is negative.

Sample size	Sample size is indicated in the figure legend and the main text. The sample size was determined based on previous published studies. No statistical methods were used to predetermine sample size.
Data exclusions	No data exclusions
Replication	All experiments were reliably reproduced in independent rats. Numbers of replicates are indicated in the figure legend and the main text.
Randomization	Rats used in this study were randomly assigned to experimental groups.
Blinding	Experimental procedure and analysis methods were constant among groups as I described in. Statistical analysis was calculated by a software automatically. We do not believe this introduced experimental bias.

Reporting for specific materials, systems and methods

We require information from authors about some types of materials, experimental systems and methods used in many studies. Here, indicate whether each material, system or method listed is relevant to your study. If you are not sure if a list item applies to your research, read the appropriate section before selecting a response.

Materials & experimental systems

n/a	Involved in the study	
x		Antibodies
x		Eukaryotic cell lines
x		Palaeontology and archaeology
	x	Animals and other organisms
x		Human research participants
x		Clinical data
x		Dual use research of concern

Methods

n/a	Involved in the study	
x		ChIP-seq
x		Flow cytometry
x		MRI-based neuroimaging

Animals and other organisms

Policy information about studies involving animals; ARRIVE guidelines recommended for reporting animal research

Laboratory animals	The experiments were performed on Wistar rats (male, 3–4 weeks; Japan SLC, Inc.)
Wild animals	none
Field-collected samples	none
Ethics oversight	All procedures were approved by the ethics review committee of the University of Tsukuba.

Note that full information on the approval of the study protocol must also be provided in the manuscript.
